# A Case Report and Literature Review of the Role of Dupilumab in the Management of Lichen Planus: Cause or Treatment?

**DOI:** 10.7759/cureus.41274

**Published:** 2023-07-02

**Authors:** Farrah A Gajraj, Jamal Zahir, Christopher Adereti, Mohamed H Gajraj

**Affiliations:** 1 Medicine, Ross University School of Medicine, Miramar, USA; 2 Surgery, Ross University School of Medicine, Miramar, USA; 3 Medicine, Coral Springs Medical Group, Coral Springs, USA

**Keywords:** lichenified drug eruption, t-cell-mediated immune response, autoimmune-mediated inflammatory dermatosis, interleukin-4, interleukin 13, drug-induced lp, atopic dermatitis, dupilumab, lichen planus

## Abstract

Lichen planus (LP) is a chronic inflammatory skin condition characterized by the six Ps (ie. purple, planar, polygonal, pruritic, plaques, and papules) often causing physical, emotional, and psychological stress for the person affected. Drug-induced LP has been described after the administration of drugs like antihypertensives, non-steroidal anti-inflammatory drugs (NSAIDs), and biologics like adalimumab and etanercept. Currently, there is a dearth of studies discussing the association between LP and dupilumab. Here, we present the case of a young adult female who developed LP 24 months after treatment with dupilumab for severe atopic dermatitis. We also conducted a review of the literature discussing the association between LP and dupilumab.

## Introduction

Lichen planus (LP) is an autoimmune-mediated inflammatory dermatosis characterized by purple, pruritic, polygonal papules, and plaques affecting the skin, mucous membranes, and nails [1,2]. Studies have shown it is a T-cell-mediated disease with a prevalence approaching 1% in the general population, often affecting middle-aged adults. In histology, LP is characterized by inflammation of the basal keratinocyte layer of the epidermis [3]. Although the exact etiology of LP remains unknown, it is thought to involve a T-cell-mediated immune response triggered by various factors, including infections (hepatitis C) and drugs (β-blockers, non-steroidal anti-inflammatory drugs (NSAIDs)) [1,4]. Recently, dupilumab, a biologic used to treat severe atopic dermatitis (AD), has been implicated in the development of LP and other skin rashes (e.g. psoriasis, lichenified eruptions) [1,5-8]. Dupilumab is a monoclonal antibody, commonly used to treat severe AD, which targets the interleukin(IL)-4 receptor alpha subunit (4Rα) found on T-helper cells type 2 (TH2); IL-4Rα normally interacts with IL-4 and IL-13 [9]. Studies have shown that IL-4Rα is a key pathway in allergic inflammation, the antagonism of which results in the modulation of the allergic inflammatory response [10]. Interestingly, dupilumab downregulates the TH2 response, leading to a TH1/TH2 imbalance, thereby increasing the rate of a TH1-mediated adaptive immune response [1]. Studies have shown that TH1-mediated immune responses result in lichenified cutaneous eruptions, like LP [1,11]. Currently, the lack of literature discussing the association of dupilumab with LP warrants a thorough investigation. In this paper, we present the case of a young adult female who developed cutaneous LP 24 months after starting dupilumab for AD.

## Case presentation

**Figure 1:**
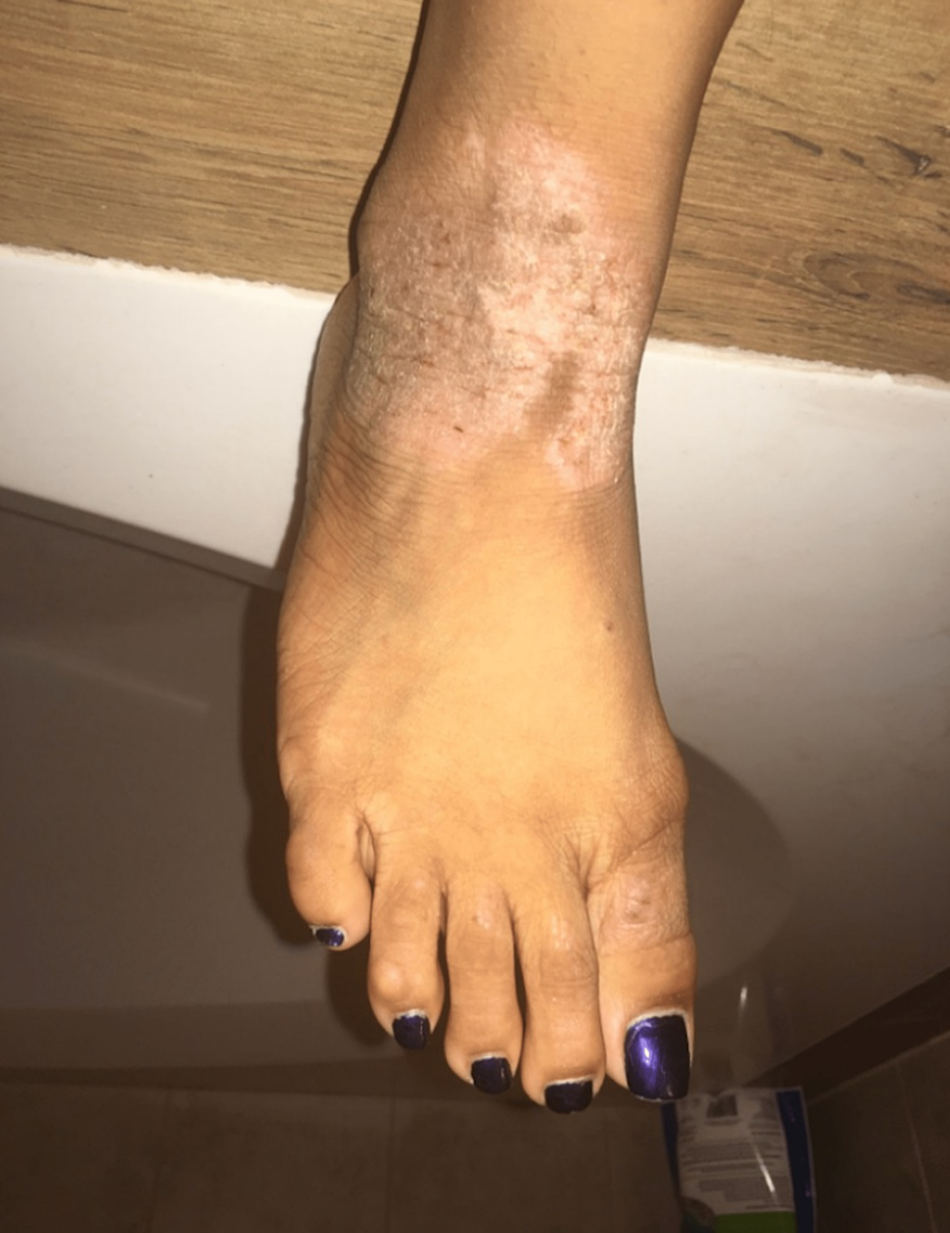
Severe Atopic Dermatitis Prior to Dupilumab Treatment

**Figure 2:**
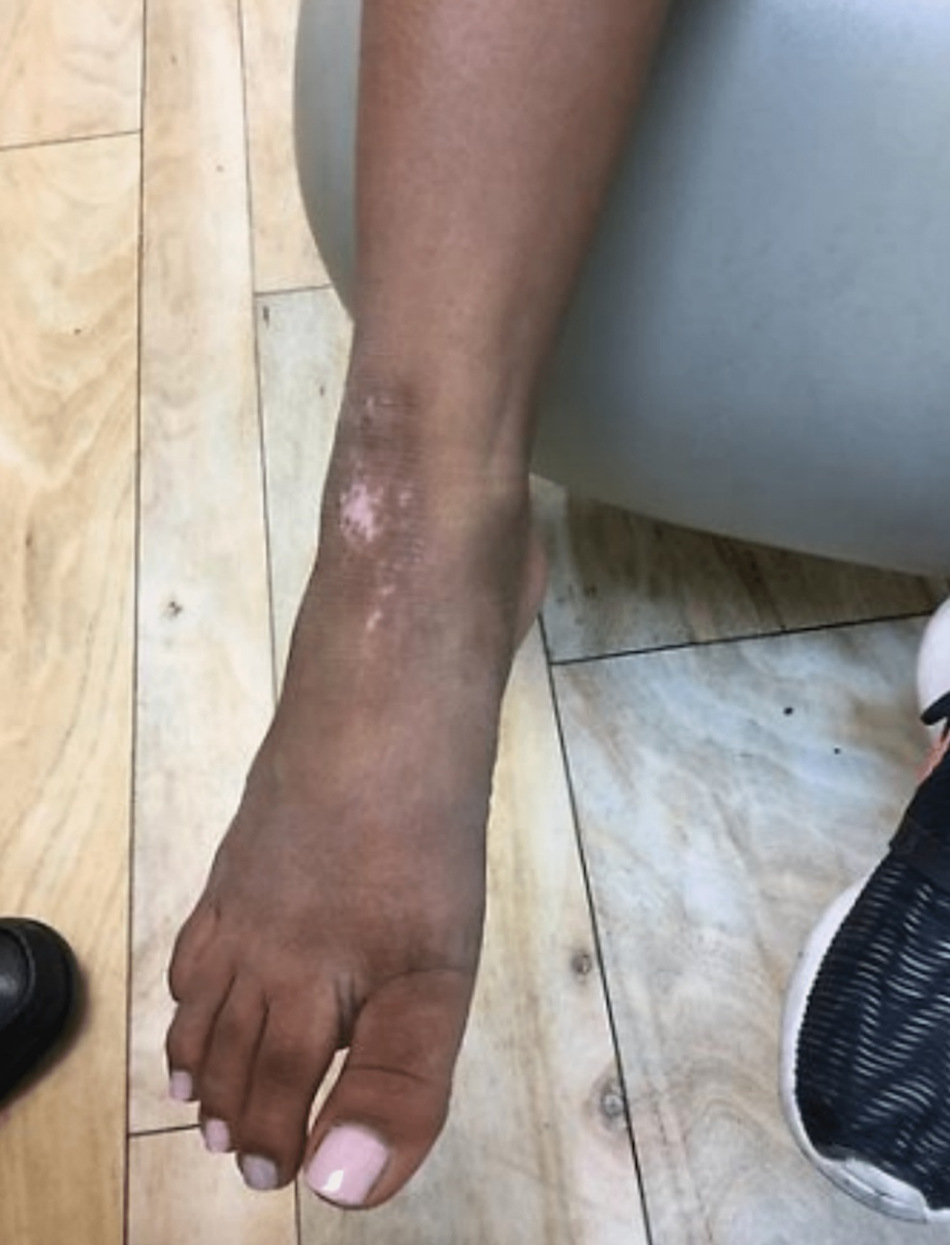
Atopic Dermatitis Six Months After Dupilumab Treatment

A 22-year-old female with a history of severe AD (Figure 1), asthma, and allergies presented to her primary physician for follow-up care after receiving treatment for her long-standing eczematous symptoms from her dermatologist. Per her medical record, previous treatment trials with topical steroids, antihistamines, and tacrolimus were given over a span of 20 years, but her condition remained refractory to therapy. It was decided to initiate the patient with 600 mg of dupilumab subcutaneously. After the first week, the dosage was reduced to 300 mg subcutaneously every other week. After six months of dupilumab treatment, the patient experienced a significant improvement in her plaques and lesions (Figure 2).

**Figure 3:**
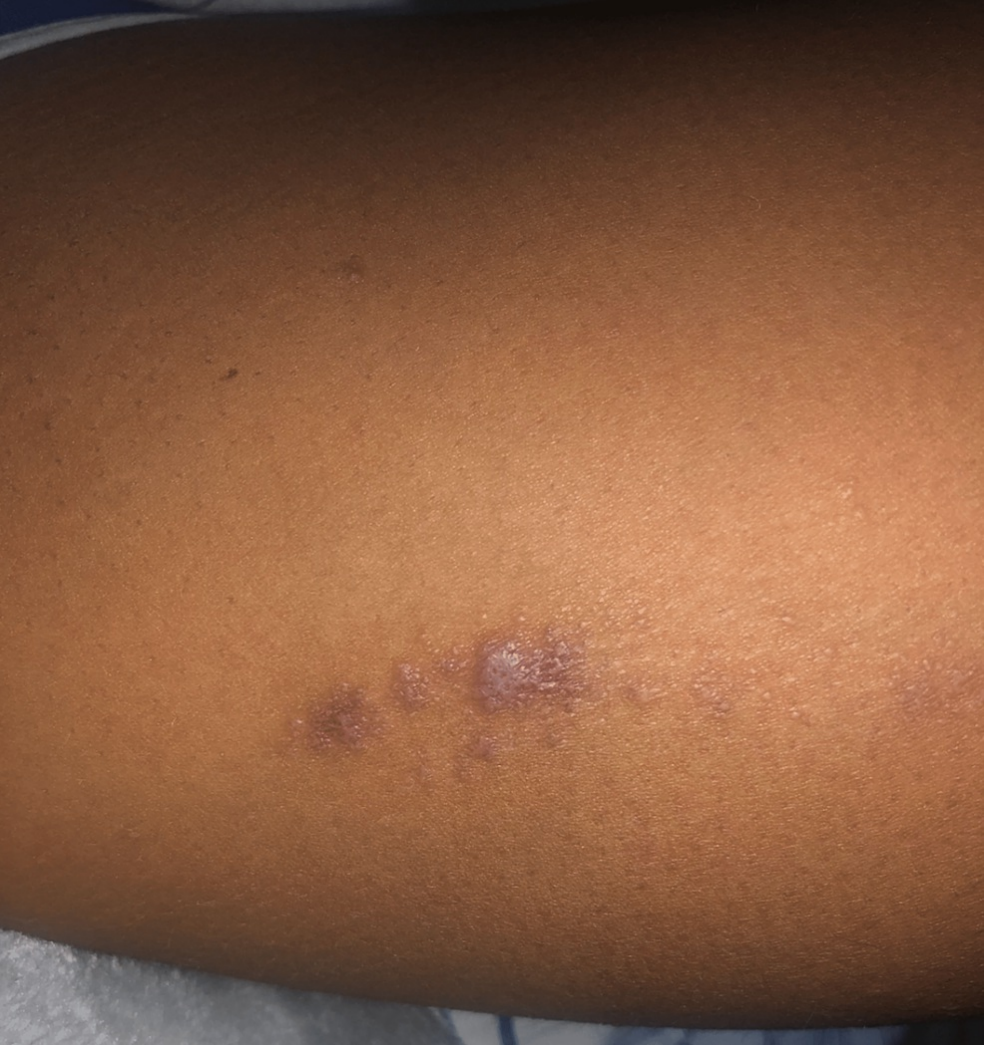
Lichen Planus Lesion on Left Inner Thigh

**Figure 4:**
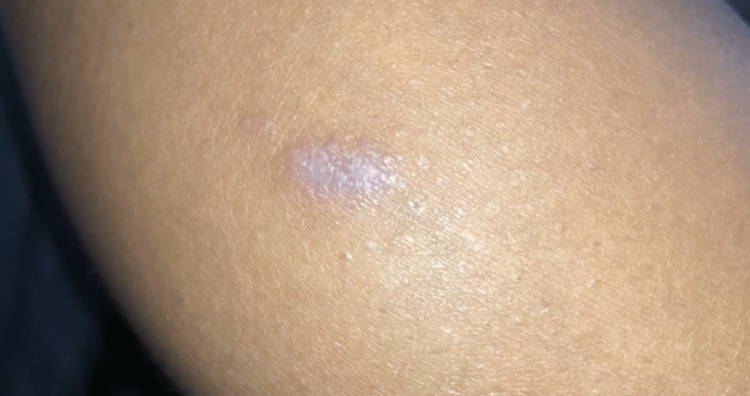
Additional Lichen Planus Lesion on Right Calf

However, after 24 months of therapy, she presented with pruritic, purple flat-topped papules distributed throughout her body, predominantly on the legs, arms, abdomen, and gluteal area (Figures 3-4). A skin biopsy was performed on a lesion from the inner left thigh, revealing a focally lichenoid infiltrate with thinning of the epidermis and an irregular jagged epidermal layer. These findings were consistent with LP and the patient was treated with a one-time dose of 40 mg of intramuscular triamcinolone. Clinically, the lesions improved 10 days after treatment.

**Figure 5:**
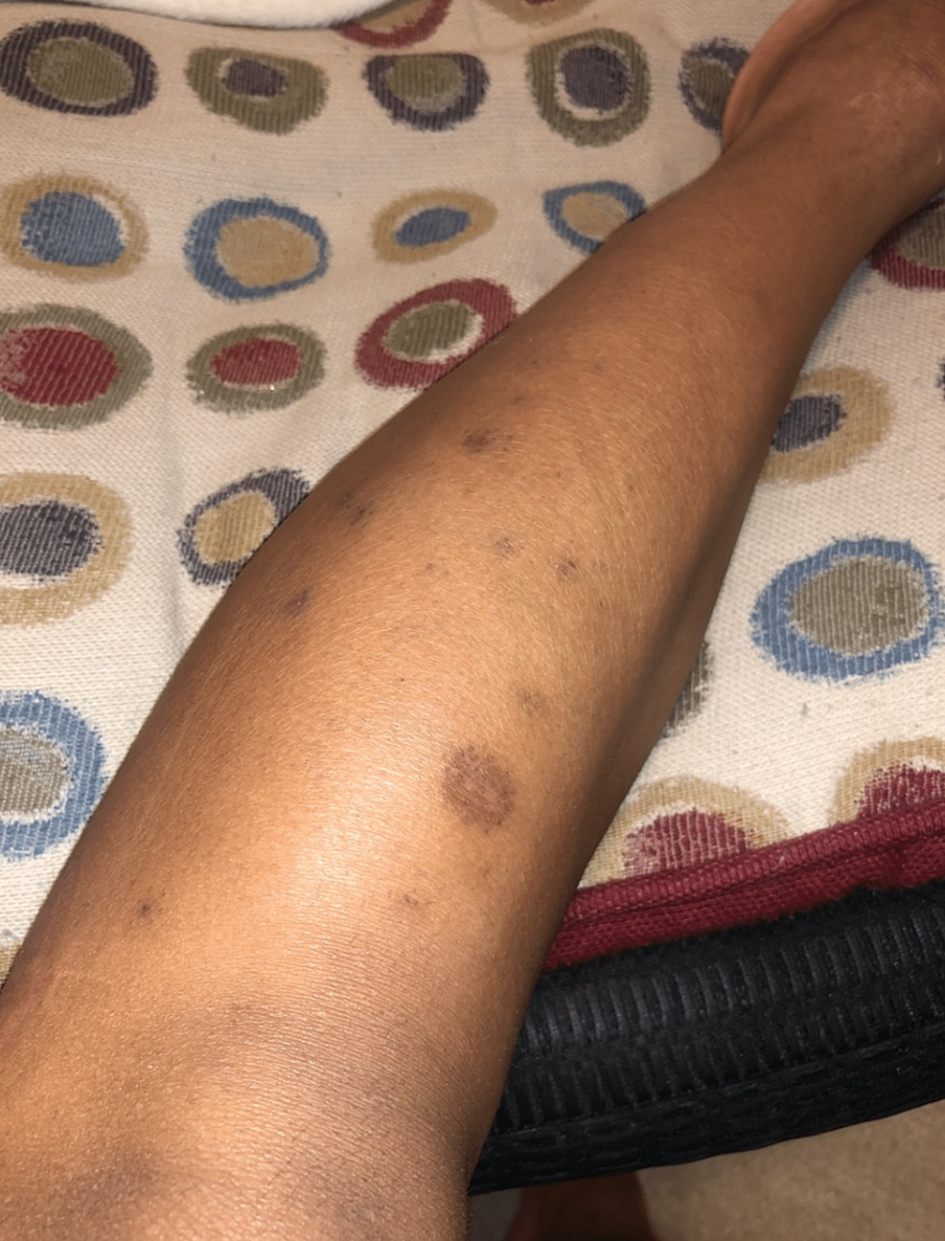
Post-Inflammatory Hyperpigmentation on the Left Leg

However, after six months, a new onset of LP lesions occurred, which were self-limited within three months. Her dermatologist did not recommend discontinuing dupilumab due to her history of severe AD, current LP resolution, and risk-benefit analysis. At the 24-month follow-up visit with her primary physician, it was revealed the patient continued to use dupilumab for AD, had residual post-inflammatory scarring and hyperpigmentation (Figure 5) in the areas previously affected by LP, and no further LP eruptions. 

## Discussion

Cutaneous LP usually presents on flexor surfaces like the wrists, antecubital region, and popliteal region and has white lacy lines overlying the lesions called "Wickham striae". LP’s association with the development of psychological problems also warrants thorough investigation about its potential causes and addressing them [12].

Recent literature has identified a possible correlation between biologics (eg. infliximab, etanercept) and the development of lichenified drug eruptions (LDE) [1,13]. In line with the present report, some other case reports have described the development of LP following the initiation of dupilumab treatment. For example, Kern et al. reported the development of cutaneous and mucosal LP in a 23-year-old woman 11 months after starting dupilumab treatment for severe AD. Treatment involved discontinuation of dupilumab and initiation of oral methylprednisolone with significant improvement in the LP at the six-week follow-up [1]. Additionally, Kim et al. reported the development of cutaneous LP 16 weeks after the initiation of dupilumab for severe AD in a 44-year-old man. Treatment involved discontinuation of dupilumab and initiation of cyclosporine with significant improvement in the plaques at the three-month follow-up [5]. These cases raise concerns about the potential of dupilumab to induce LP or exacerbate pre-existing LP in certain individuals. The exact mechanisms underlying the development of LP after initiation of dupilumab remain unclear and should be a topic of investigation in future studies. On the other hand, Halevy et al. reported that patients with LDE experienced intermittent relapse and resolution despite continued treatment with an offending agent [4]. This underscores the notion that certain LDE may actually subside even if the causative drug continues to be administered. Hence, it's plausible that in certain instances of LDE, discontinuation of the offending drug isn't always required, particularly if an abrupt cessation could pose a risk to the patient's health [4]. Thus, although our patient showed resolution of LP at the 24-month follow-up, this does not exclude the possibility of another LP relapse in the future, despite continued treatment with dupilumab.

While the previous reports discussed a potential correlation between the initiation of dupilumab and the development of LP, other studies described the treatment of LP with dupilumab [14-16]. Pousti et al. described the case of a 52-year-old man who progressively developed biopsy-proven LP over a six-month period. He failed initial treatment with halobetasol ointment, tacrolimus ointment, and oral prednisone taper. Next, he also failed treatment with acitretin. Due to the failure of common United States Food and Drug Administration (FDA) treatment, off-label use of dupilumab was started with remarkable improvement in LP at the three-month follow-up. Their patient did not have a history of AD and his workup for hepatitis C, syphilis, and HIV was all negative [16]. Interestingly, Kazemi et al. presented the case of a 92-year-old female with biopsy-proven LP developed over a two-year period. Initial management with oral prednisone resulted in transient relief. For that reason, dupilumab 600 mg initially followed by 300 mg biweekly resulted in complete resolution in LP lesions at the two-month follow-up [17].

Although not the same as LP, LP pemphigoides (LPP) is a similar skin eruption characterized by a combination of LP and bullous pemphigoid. Li et al. reported the case of a 69-year-old man with a 20-year history of mucosal LP, who developed red papules and plaques over a one-year period and was diagnosed with LPP. He failed treatment with IV prednisolone 50 mg daily and halometasone triclosan ointment. After minimal improvement with the initial regimen, dupilumab was then added with clinical improvement on day three and significant improvement at the two-month visit [18]. Additionally, Ch’en et al. described the case of an 18-year-old man with a history of AD who acutely developed biopsy-proven LPP. Initial management included oral dexamethasone 8 mg daily after which the LPP lesions improved at the 10-week follow-up. However, six months later, there was a recurrence of worsening LPP. At this point, dupilumab treatment was initiated with complete resolution of LPP at 15 weeks. He then discontinued dupilumab with no recurrence of LPP at the four-month follow-up [19].

Along with LP and LPP, the initiation of dupilumab has also been correlated with the development of psoriasiform dermatitis. For example, Fowler et al. reported a case of a 54-year-old male with a history of severe AD who was treated with dupilumab. He had marked improvement in his severe AD after one month; however, he developed biopsy-proven psoriasiform dermatitis eight months after treatment with dupilumab. Initial treatment involved discontinuation of dupilumab with marked improvement of psoriasiform dermatitis at his two-month follow-up appointment [8]. 

Next, Fowler et al. presented a case of a 49-year-old female with a history of moderately severe AD treated with dupilumab after an initial course of topical corticosteroids and phototherapy failed. After 18 months of dupilumab treatment, she developed psoriasiform dermatitis treated with clobetasol ointment twice daily which resulted in significant improvement [8]. Albader et al. discussed a case of a 28-year-old female with a history of AD since childhood. She failed initial treatment with cyclosporine and methotrexate and was started on dupilumab. After four months of dupilumab treatment, she developed an erythematous facial rash extending to the neck. Improvement of the rash was noted with hydroxyzine and topical mometasone furoate treatment after two days of use. Workup for systemic lupus erythematosus (SLE) was negative including negative anti-nuclear antibody (ANA) and normal erythrocyte sedimentation rate (ESR) [7].

These findings support the hypothesis that blocking IL-4 and IL-13 signaling pathways by dupilumab can modulate the immune response and may treat LP and LPP [20]. The development of LP in some patients receiving dupilumab may involve complex interactions between immune dysregulation, genetic susceptibility, patient heterogeneity, comorbid conditions, and the modulation of cytokine signaling pathways. The possibility of dupilumab not only treating but causing LP necessitates further investigation in future studies. Therefore, a careful risk-benefit analysis of dupilumab treatment should be considered. Close monitoring for potential adverse effects, including the development or exacerbation of LP, is essential during dupilumab therapy. Due to the clinical and histopathological similarities between LDE and idiopathic LP, it can be challenging to definitively determine if a drug has caused LP [4]. This warrants further investigation with larger studies and a more diverse population. While there is evidence of successful outcomes with dupilumab in LP patients, caution is necessary due to conflicting reports and the possibility of dupilumab-induced LP. Future research efforts should aim to increase sample size, elucidate the underlying mechanisms of LP development, and optimize the use of dupilumab.

The discrepancies reported may be attributed to several factors, including patient heterogeneity, variations in disease characteristics, and differences in treatment regimens. LP itself encompasses various subtypes and individual responses to treatment can vary. The interplay between dupilumab, genetic predisposition, immune dysregulation, and co-existing conditions may influence the outcomes observed in LP patients. Table 1 highlights the key findings from the relevant articles discussing treatment with dupilumab.

**Table 1:** Summary of Key Findings from Cases of Patients Undergoing Treatment with Dupilumab M: Male; F: Female; AD: Atopic Dermatitis; N/A: Not Applicable; NS: Not Specified

Author	No. of cases (n)	Sex (M/F)	Patient age (in years)	Pre-existing dermatologic condition	Adverse Effect of Dupilumab	Time to development of adverse effect following Dupilumab	Treatment of Adverse Effect	Clinical Outcome
Kim et al. [5]	1	M	44	AD	Lichen Planus	16 weeks	Termination of dupilumab	Clinical improvement at 3 months
Kern et al. [1]	1	F	23	AD	Lichen Planus	11 months	Termination of dupilumab; Oral Steroid	Clinical Improvement at 10 days
Li et al. [18]	1	M	69	Non-bullous Lichen Planus Pemphigoides	None	N/A	N/A	Significant improvement at 2 months
Ch’en et al. [19]	1	M	18	Lichen Planus Pemphigoides	None	N/A	N/A	Complete resolution at 15 weeks
Pousti et al. [16]	1	M	52	Lichen Planus	None	N/A	N/A	Clinical improvement at 3 months
Kazemi et al. [17]	1	F	92	Lichen Planus	None	N/A	N/A	Clinical improvement at 2 months
Fowler et al. [8]	2	M	54	AD	Psoriasiform Dermatitis	8 months	Termination of dupilumab	Clinical improvement at 2 months
		F	49	AD	Psoriasiform Dermatitis	18 months	Clobetasol Ointment	Clinical Improvement (Duration NS)
Albader et al. [7]	1	F	28	AD	Face and neck rash	4 months	Hydroxyzine; mometasone furoate cream	Clinical improvement at 2 days

## Conclusions

Although many studies show promising evidence that dupilumab may treat LP, other studies have shown that dupilumab may actually induce LP or other inflammatory dermatoses. It thus remains unclear whether dupilumab induces or cures LP. Due to the lack of studies and clear evidence guiding clinicians on dupilumab’s role in the treatment or induction of LP, future researchers should conduct studies with large sample sizes and a diverse population of patients. This will result in clear risk stratification about whether dupilumab can be safely prescribed for patients.
